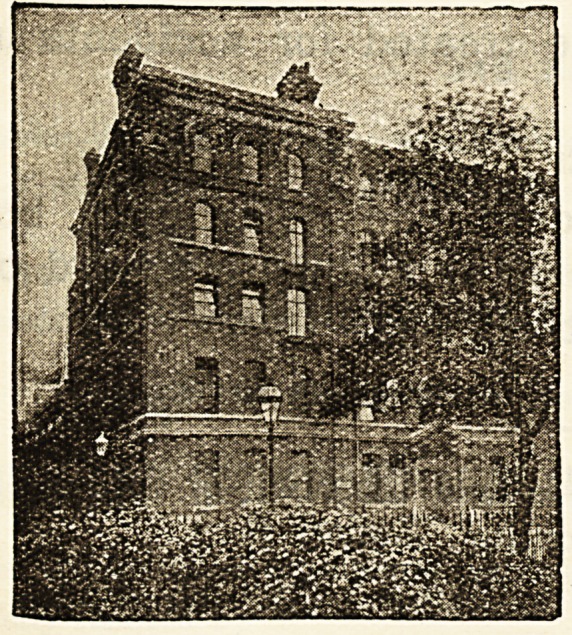# Christmas Appeal Supplement

**Published:** 1904-12-10

**Authors:** 


### CHRISTMAS APPEAL SUPPLEMENT.

No. 950. Vol. XXXVII. THE HOSPITAL Saturday, Dec. 10, 1904.

### Mount Vernon Hospital for Consumption and Diseases of the Chest,

### HAMPSTEAD AND NORTHWOOD.

**President—The MARQUIS OF ZETLAND, K.T.**

The Committee of Management most earnestly plead for greater financial support to enable them to open the remaining 120 Beds in the Hospitals at Hampstead and Northwood.

The urgent need of these Beds is amply demonstrated in the fact that there are now nearly 300 accepted applicants waiting for admission. Meanwhile 120 Beds are lying idle, and hundreds of applicants have to be refused altogether, as it is useless to add to the already long waiting list.

Until the present liabilities of the Hospital—nearly £15,000—are liquidated, it will not be possible to open more than the present number of Beds.

**£1,000 will endow a Bed. £500 will endow a Cot.**

Bankers: MESSRS. HOARE, 37 Fleet Street, E.C.

CHEQUES MAY BE SENT TO—

**Offices: 7 FITZROY SQUARE, W. WILLIAM J. MORTON, Secretary.**

### East London Hospital for Children

### AND DISPENSARY FOR WOMEN.

### SHADWELL, E.

**Seaside Branch: "Princess Mary Convalescent Home," Aldwick Road, Bognor, Sussex.**

**Patrons:**

HIS MAJESTY THE KING. HER MAJESTY QUEEN ALEXANDRA.

**President:**

HIS GRACE THE DUKE OF PORTLAND, K.G.

**Chairman:**
COLONEL NEEDHAM.

**Vice-Chairman:**
THE LORD CASTLETOWN.

**Treasurer:**
KENNETH PRESCOTT, Esq.

THE HOSPITAL was founded in 1868 and has 135 beds—107 at SHADWELL and 28 at the Seaside Branch, BOGNOR.

It is FREE—UNSECTARIAN UNENDOWED.

The ANNUAL COST of MAINTENANCE (averages)—

| | |
|---|---|
| At the Hospital ... ... ... ... ... ... ... ... | £9,000 |
| At the Seaside Branch ... ... ... ... ... ... ... | 900 |
| | £9,900 |
| The only ASSURED INCOME (derived from sums invested on account of Special Cots, Legacies, &c.) is ... ... ... | 975 |
| Leaving ... ... ... ... | £8,925 |

to be obtained Annually from *VOLUNTARY CONTRIBUTIONS.*

ANNUAL SUBSCRIPTIONS and DONATIONS for MAINTENANCE purposes are earnestly asked for.

**A Special Appeal** is made for

### £4,000

to enable the Committee to clear off present liabilities and to meet maintenance expenses to the 31st December. It is worthy of remark that until last year the Hospital had never borrowed money.

Cheques should be crossed "Union of London and Smiths' Bank, Ltd.," and made payable to

December 1904. **THOMAS HAYES, Secretary.**

### ST. THOMAS'S HOSPITAL,

**Thames Embankment, S.E.**

**SERVES A VERY LARGE POOR POPULATION ON THE SOUTH OF THE THAMES.**

There are 561 beds for the ABSOLUTELY POOR.
There are 42 Beds in St. Thomas's Home for cases who can pay a moderate amount.

IN-PATIENTS.—6,774 cases were treated in 1903.
" 644 were treated in St. Thomas's Home in 1903.
OUT-PATIENTS.—65,703 were treated in 1903.

| | |
|---|---|
| The Hospital Expenditure on Maintenance and special repairs amounted in 1903 to ... | **£61,442.** |
| The Income from Endowments, paying patients, etc, amounted to ... ... ... ... | **£55,858.** |
| Capital Expenditure on New Buildings and Structural Improvements ... ... ... | **£33,714.** |

It is thus seen that a considerable amount in 1903 was required from **VOLUNTARY CONTRIBUTIONS** to maintain the Hospital in its efficiency.

To meet the cost of Extensive Building Improvements £7,457 Stock was sold last year, and more must be sold.

Contributions to be sent to the Treasurer, J. G. WAINWRIGHT, Esq., at the Hospital, or to
G. Q. ROBERTS, Secretary.

***Applications for admission to St. Thomas's Home for Paying Patients are to be sent to the Steward.***

**NORTH-EASTERN**

### HOSPITAL FOR CHILDREN,

**HACKNEY ROAD, BETHNAL GREEN, E.**

**Patron—H.M. QUEEN ALEXANDRA.**

### Second Largest Children's Hospital in London.

### 114 BEDS.

**Must Close Half its Wards at end December**

unless sufficient assistance can be obtained meanwhile.

### IMMEDIATE HELP NEEDED.

**250** Accident and Emergency Cases Weekly. **64,000** Out-patient Attendances Annually.

BANKERS—BARCLAY'S, Lombard Street. T. GLENTON-KERR, Secretary.

### INDEX TO ADVERTISEMENTS.

| | PAGE |
|---|---|
| Association for Oral Instruction | 24 |
| Birmingham General Hospital | 6 |
| Bournemouth National Sanatorium for Consumption | 6 |
| British Orphan Asylum | 26 |
| Cancer Hospital | 25 |
| Chelsea Hospital for Women | 10 |
| Children's Hospital, Great Ormond Street Back Cover | 28 |
| City of London Lying-in Hospital | 25 |
| Destitute Children's Dinner Society | 9 |
| East London Hospital for Children ....Cover | 2 |
| Field Lane Refuges ....Cover | 27 |
| Great Northern Central Hospital | 23 |
| Hampstead Hospital | 25 |
| Hospital and Home for Incurable Children | 26 |
| Hospital for Invalid Gentlewomen | 9 |
| Hospital for Women, Soho Square | 8 |
| Irish Distressed Ladies Fund | 24 |
| King's College Hospital | 7 |
| London Fever Hospital | 26 |
| London Hospital | 8 |
| London Homœopathic Hospital | 5 |
| London Lock Hospital | 24 |
| London Orphan Asylum | 25 |
| Mary Wardell Convalescent Home ......Cover | 27 |
| Metropolitan Hospital | 5 |
| Metropolitan Convalescent Institution..Cover | 27 |
| Miller Hospital | 26 |
| Mount Vernon Hospital | Front Cover |
| North-Eastern Hospital for Children | 3 |
| North-West London Hospital | 25 |
| Orphan Working School | 24 |
| Paddington Green Children's Hospital | 8 |
| Poplar Hospital | 7 |
| Queen Charlotte's Lying in Hospital | 23 |
| Queen's Jubilee Hospital | 8 |
| Royal Free Hospital | 10 |
| Royal London Ophthalmic Hospital | 9 |
| Royal Maternity Charity | 24 |
| St. George's Hospital | 9 |
| St. Mary's Hospital | 4 |
| St. Saviour's Hospital | 7 |
| St. Thomas's Hospital | 3 |
| Samaritan Free Hospital | 26 |
| University College Hospital | 10 |
| Victoria Hospital for Children | 9 |
| West End Hospital | 5 |
| Wolverhampton Royal Orphanage ......Cover | 27 |

### ST. MARY'S HOSPITAL,

### PADDINGTON, W.

THE resources of this Charity are mainly spent in the relief of the Sick Poor of Paddington, Kensington, and Marylebone.

These three parishes, among the richest in London, contain, nevertheless, a population exceeding **100,000 souls living in a state of poverty and distress scarcely paralleled, certainly not exceeded, in the East End.**

Thus engaged, in the midst of affluence, in relieving the sufferings of thousands of the poorest poor, the Hospital is thought by many, from its well-to-do surroundings, to be liberally supported.

Nothing could be further from the fact. For years past, owing to the inadequacy of subscriptions, Legacies which should have been invested to create an income have had to be spent to provide for current expenses.

**The NEED OF FUNDS IS MOST URGENT, and the Board of Management earnestly ask for assistance.**

**It costs £30,000 a year to maintain the 281 Beds, in which 4,000 Patients are treated.**

**The Hospital is Free, and no urgent case is ever refused admission.**

THOMAS RYAN, Secretary.

### London Homœopathic Hospital,

**GREAT ORMOND STREET, BLOOMSBURY, W.C.**

### A GENERAL HOSPITAL

For Men, Women, and Children, including Special Departments for Diseases of Women, Diseases of the Skin, Diseases of the Eye, Diseases of the Throat, Nose and Ear, Diseases of Children, and Diseases of the Nervous System; also for Electricity.

THE urgent need of this Hospital is an increase of **ANNUAL SUBSCRIPTIONS.** The sum needed for EACH DAY'S MAINTENANCE is **THIRTY GUINEAS,** while the income FOR EACH DAY is under **TWENTY-ONE GUINEAS.** Who will make one of the **365 NEW ANNUAL SUBSCRIBERS of TEN GUINEAS** each required to prevent curtailing the work of the Institution, and enable it to regain the position it has so long held among Metropolitan Hospitals of "paying its way year by year." It is an exceedingly active Hospital, comprising not only general medicine, but various special departments, together with a large and varied surgical work, also an Electrical Department for Roentgen Rays and the treatment of Lupus. A contemporary says:—"The Homœopathic Hospital has many claims upon public support. It has been described as one of the most beautiful Hospitals in London, and it is certainly one of the most economically conducted." It has been over fifty years in operation, and has tended many thousands of patients. Last year **1,145 In-Patients** were relieved, and the **Out-Patients** consultations numbered **43,289.** The Hospital is open to inspection every week-day between Ten and Four, when Visitors will receive a hearty welcome.

Cheques should be crossed "THE LONDON AND WESTMINSTER BANK, or the UNION OF LONDON and SMITHS BANK," and addressed to the SECRETARY, London Homœopathic Hospital, Great Ormond Street, London, W.C.

---

### METROPOLITAN HOSPITAL,

**KINGSLAND ROAD, N.E.**

Patron—HIS MAJESTY THE KING.

*Chairman*—The Right Hon. LORD HOWARD DE WALDEN.

*President*—The Right Hon. The LORD MAYOR.

*Treasurers*—The Right Hon. LORD HILLINGDON, Right Hon. LORD BATTERSEA, LEOPOLD DE ROTHSCHILD, Esq.

*Hon. Secretary* ... ... SIR E. HAY CURRIE.

**There are 111 Beds available for In-patients. The Charity has an endowment of only £434 a year, and is dependent entirely upon Voluntary Contributions.**

**In-patients, 1903 - - - - - - 1,270**

**Out-patients „ (attendances) - - 107,633**

**The Committee most urgently Appeal for Help.**

Bankers—GLYN, MILLS and CO.; LLOYDS BANK, Ltd. CHARLES H. BYERS, Secretary.

---

### WEST END HOSPITAL

For DISEASES OF THE NERVOUS SYSTEM, PARALYSIS, and EPILEPSY,

**73 WELBECK STREET, LONDON, W.**

Patroness—HER MAJESTY THE QUEEN.

Maintains fifty Cots for little Paralysed and Nervous Children, who are admitted from all parts. This Hospital is totally without endowment.

FUNDS URGENTLY NEEDED to meet the current expenditure. Cheques and postal orders may be crossed "Sir Samuel Scott, Bart. and Co."

H. C. WILLOCK, Honorary Treasurer.
ALFRED J. WISE, Secretary.

### NATIONAL SANATORIUM FOR CONSUMPTION

### AND DISEASES OF THE CHEST,

### BOURNEMOUTH.

(Founded 1855.)

**OPEN-AIR TREATMENT.**

Patron—HIS MAJESTY THE KING.

Vice-Patrons { The Right Hon. the EARL OF MOUNT-EDGCUMBE, G.C.V.O., The Right Hon. the LORD WIMBORNE.

President—The Right Hon. the EARL OF ELDON.

Affords Relief to the Necessitous Poor by a Stay in a Salubrious Climate (Three Acres of Grounds.)

**£1,500 MOST URGENTLY NEEDED BEFORE DEC. 31st, TO MEET PRESENT LIABILITIES, viz.:—**

**£800 to complete the sum of £2,700 for the New Extension of the Sanatorium.**
**£200 to furnish the New Wards, Nurses' Quarters, &c.**
**£500 for Ordinary Current Expenses.**

In addition to the above, NEW ANNUAL SUBSCRIPTIONS to the amount of **£500** must be obtained to maintain the ADDITIONAL Patients for whom accommodation is being provided, otherwise part of the Institution must be closed.

**1905** being the **JUBILEE YEAR** of the National Sanatorium, the Committee most earnestly desire to celebrate the event by extinguishing the present heavy debt.

Further particulars concerning this Charity and copy of the Annual Report will be gladly forwarded upon application to the Secretary, A. G. A. MAJOR, at Bournemouth, or to the London Secretary, 32 Sackville Street, Piccadilly, W.

### BIRMINGHAM GENERAL HOSPITAL.

FOUNDED 1766. NEW BUILDING OPENED 1897.

### SUPPORTED BY VOLUNTARY CONTRIBUTIONS.

**THE ANNUAL EXPENDITURE EXCEEDS } £25,000** | **INCOME FROM INVESTMENTS £6,000**; **ANNUAL SUBSCRIPTIONS ... £8,000**

**346 Beds. 329 available.**

During the year 1903 there were admitted 5,336 In-Patients and 64,926 Out-Patients (a total of 70,292 cases), of which 4,031 In- and 42,396 Out-Patients were admitted **without Ticket.**

**The Jaffray Branch at Gravelly Hill** (56 beds) receives the more chronic cases from the Wards, and so enables a larger number to be admitted to the parent Institution.

**Separate Subscription List of £350. Expenditure £2,500.**

**NEW AND INCREASED ANNUAL SUBSCRIPTIONS ARE URGENTLY NEEDED**

to meet the increased expenditure required for the larger number of beds now available, and will be thankfully received by

HOWARD J. COLLINS, House Governor.

Under the Patronage of H.R.H. Princess Christian.

### St. Saviour's Hospital for Ladies of Limited Means,

**OSNABURGH ST., N.W.**

**Terms from One Guinea to £2 12s. 6d. weekly.**

For particulars apply to the Sister-in-Charge.

**ANNUAL SUBSCRIPTIONS & DONATIONS**

**ARE URGENTLY NEEDED TO CARRY ON THIS WORK, THE USEFULNESS OF WHICH IS YEARLY INCREASING.**

The Hospital is under the honorary Medical Supervision of several eminent Physicians and Surgeons, and is especially adapted for the class for whom it is intended.

Hon. Sec., CYRIL COBB, Esq.

Telegraphic Address: "SORELLA, LONDON." | Telephone: 772, KING'S CROSS.

### King's College Hospital,

**PORTUGAL STREET,**

**LINCOLN'S INN FIELDS, W.C.**

Patron: His Majesty the King.

**TO MEET IMMEDIATE REQUIREMENTS** Contributions in aid of the **CURRENT EXPENSES** of the Hospital are earnestly requested.

**Donations and new or increased Annual Subscriptions will be thankfully received by the Treasurer, CHARLES AWDRY, Esq., or by**

H. S. TUNNARD, Secretary.

### POPLAR HOSPITAL FOR ACCIDENTS.

### REASONS FOR HELPING.

1. Situated amongst a teeming population of poor hard-working people in a district that may be called the "workshop" as well as the "Port" of London.
2. Accidents treated at the rate of NINE an hour for every day of every year.
3. Nine miles of men standing side by side treated for accidents in one year.
4. Working men give £600 a year in subscriptions and donations.
5. No endowment—but has never been in debt, and never shall be. If subscriptions fall off the work will be curtailed, and ward or wards closed. This would cause great misery.
6. No Letters required.

CHAIRMAN—Hon. SYDNEY HOLLAND, 44 Bryanston Square,

SECRETARY—Lt.-Col. FENERAN, Poplar Hospital, Poplar, E.

**Old Linen very valuable.** **Game a great treat.**

### LONDON HOSPITAL, E.

**The Committee appeal for £60,000 a-year from VOLUNTARY CONTRIBUTIONS**

FOR THE MAINTENANCE OF THIS GREAT CHARITY.

The number of IN-PATIENTS treated in 1902 was 13,160
„ OUT-PATIENTS „ „ 162,147
The average number daily in the Wards is 682.

**FUNDS ARE VERY URGENTLY NEEDED TO CARRY ON THE WORK EFFICIENTLY.**

Thoroughly-Trained Private Nurses to be had immediately on application to the Matron.

HONBLE. SYDNEY HOLLAND, *Chairman.* E. W. MORRIS, *Secretary.*

### THE QUEEN'S JUBILEE HOSPITAL,

**EARL'S COURT, S.W.**

(Founded in 1887 as a permanent Memorial of our late Most Gracious Queen.)

**Supported entirely by Voluntary Contributions.**

**Funds urgently needed,** not only for treating the ever-increasing numbers of sick poor, who attend free of charge, but to enable the Committee to extend the present building.

The Councils of King Edward's Fund, Hospital Sunday Fund, and the Charity Organisation Society, consider it necessary to provide better and greater accommodation.

Any Donation or Subscription, no matter how small, will be thankfully received by the SECRETARY at the Hospital, from whom a report can be obtained.

### Paddington Green Children's Hospital,

PADDINGTON GREEN, LONDON, W.

**(With Convalescent Home for 16 Children at Fair View, Slough, Bucks).**

SUPPORTED ENTIRELY BY VOLUNTARY CONTRIBUTIONS.

*Free to the Sick Children of the Poor without Letter of Recommendation.*

The Hospital provides **46** Cots. Nearly **1,000** Out-patients Weekly.

**FUNDS ARE URGENTLY NEEDED.**

The Hospital owes its Bankers **£2,000,** and there is a debt of **£500** on the Convalescent Home.

DOUGLAS OWEN, Esq., Chairman, 53 Drayton Gardens, S.W.
GEORGE HANBURY, Esq., Treasurer, 28 Prince's Gate, S.W.

### The Hospital for Women,

SOHO SQUARE, LONDON, W.

*FOUNDED 1842.*

**Incorporated by Royal Charter, 1887.**

**Patron**—HER MAJESTY QUEEN ALEXANDRA.

**President**—THE EARL OF SHAFTESBURY. **Treasurer**—F. A. BEVAN, ESQ.

THE Hospital for Women was the first establishment in this or any other country exclusively for the treatment of diseases peculiar to Women.

This National Institution is *entirely dependent upon Voluntary Contributions,* which are **most urgently needed.**

In addition to the Free Wards, the New Wing, opened in 1869, is available for those able to contribute a weekly sum towards their maintenance.

**BANKERS—Messrs. Barclay & Co., Limited, 54 Lombard Street, E.C., and 1 Pall Mall East, S.W.**

DAVID CANNON, Secretary.

### ST. GEORGE'S HOSPITAL, S.W.

**Patron—THE KING'S MOST EXCELLENT MAJESTY.**
**President—H.R.H. THE PRINCE OF WALES, K.G.**

### ADDITIONAL CONTRIBUTIONS EARNESTLY SOLICITED.

The ordinary expenditure exceeds the ordinary income by upwards of **£15,000.**
In-Patients and Out-Patients treated annually about **36,000.**

Secretary, C. L. TODD. Treasurers { LORD WINDSOR. A. WILLIAM WEST, Esq.

### CENTENARY APPEAL.

### ROYAL LONDON OPHTHALMIC HOSPITAL

### CITY ROAD, E.C.

For 100 years the ROYAL LONDON OPHTHALMIC HOSPITAL (known to the suffering poor as Moorfields Eye Hospital) has gratuitously relieved the patients who come to it from all parts of the Kingdom.

The Committee earnestly appeal for generous help to enable them to carry on the work of this National Charity.

Over **100** In-patients, and over **400** Out-patients, are relieved at the Hospital daily.

ROBERT J. BLAND, *Secretary.*

**Patroness—Her Most Gracious Majesty THE QUEEN.**

### The Hospital for Invalid Gentlewomen

**During Temporary Illness.**

### 90 HARLEY STREET.

### LADIES ARE RECEIVED FOR GRATUITOUS MEDICAL AND SURGICAL TREATMENT.

Terms, including Board and Nursing, from £1 5s. to £2 5s. 6d. per week. For full particulars, apply to Miss TIDY, Lady Superintendent.

### VICTORIA HOSPITAL FOR CHILDREN,

### TITE STREET, CHELSEA, S.W.

CONVALESCENT HOME: BROADSTAIRS.

**Funds are Urgently Needed for the Maintenance of the Hospital and Convalescent Home.**

The reliable Income on which the Committee have to depend is under £3,000, and the Annual Expenditure exceeds £8,000.

The Hospital is doing its utmost to alleviate the sufferings of the 53,000 children who annually seek relief at the Hospital. 32 beds are closed for want of Funds.

**£12,000 is Required to Pay off the Debt on the New Buildings, which contain 104 Beds.**

Annual Subscriptions and Donations will be gratefully acknowledged by H. G. EVERED, Secretary.

### THE DESTITUTE CHILDREN'S DINNERS SOCIETY.

ESTABLISHED 1866.

**Patron—HER MAJESTY THE QUEEN.**

**President**—The BARONESS BURDETT-COUTTS.
**Treasurer**—LORD KINNAIRD. **Secretary**—H. MORTON CARR, Esq.
**Bankers**—Messrs. BARCLAY & CO., 1 Pall Mall East.

The work of this Society extends to all parts of London. It has 37 dining rooms open in the poorer districts, and provides good meat dinners to thousands of poor children attending schools. £150 per month is required to carry on this useful and benevolent work.

Funds are urgently needed, and Subscriptions, Donations, Legacies, &c., will be thankfully received by the Bankers, Treasurer, or the Secretary, 22 Lavender Gardens, Lavender Hill, S.W.

Open FREE to the Sick Poor without LETTERS OF RECOMMENDATION; POVERTY and SUFFERING being the ONLY PASSPORTS required.

The Hospital annually admits into its Wards over 2,500 Poor Sick Persons, besides administering advice and medicine to about 35,000 Out-Patients.

### ROYAL FREE HOSPITAL,

**GRAY'S INN ROAD, W.C.**

**Under the Patronage of**

THE KING,

THE QUEEN.

The Annual Expenditure is over £14,000, while the reliable Income does not exceed £4,000.

Contributions will be thankfully received by the Bankers, LLOYDS BANK, LIMITED, Holborn Circus, E.C., or at the Hospital by

CONRAD W. THIES,

Secretary.

### Chelsea Hospital for Women

### FULHAM ROAD, S.W.

**Patron—HER MAJESTY THE QUEEN.**

| | |
|---|---|
| Chairman ... ... ... ... ... | THE LORD GLENESK. |
| Vice-Chairman ... ... ... ... | T. DYER EDWARDES, Esq., J.P. |
| Treasurer ... ... ... ... ... | HENRY E. WRIGHT, Esq. |

The Hospital contains 50 beds (774 In-Patients during 1903), for the treatment of gentlewomen in reduced circumstances and respectable poor women suffering from those distressing diseases to which the female sex in particular is liable.

**Important alterations** have been carried out in order to give patients the benefits of the **latest medical science**, and DONATIONS and NEW ANNUAL SUBSCRIPTIONS are greatly needed.

The CONVALESCENT HOME, West Hill, St. Leonards-on-Sea, with 22 beds, is invaluable to the patients, and is not restricted to Hospital cases. Number of Patients received during 1903 277, of whom 180 were sent from the Hospital.

HERBERT H. JENNINGS, *Secretary.*

### NORTH LONDON OR UNIVERSITY COLLEGE HOSPITAL,

Patron—THE KING.] **GOWER STREET, W.C.** [President—The DUKE OF BEDFORD.

**A GENERAL HOSPITAL WITH SPECIAL DEPARTMENTS.**

**NEARLY 50,000 PATIENTS TREATED ANNUALLY.**

| | |
|---|---|
| **Annual Expenditure** ... ... ... ... ... | **£22,000** |
| **Income from all sources** ... ... ... ... | **9,000** |
| **ANNUAL DEFICIT** to be met by Voluntary Contributions | **£13,000** |

**FUNDS URGENTLY NEEDED.**

NEWTON H. NIXON, *Secretary.*

### OUR CHRISTMAS SUPPLEMENT.

### The Guest-House.

### WHERE ALL MAY ENTERTAIN GUESTS.

The voluntary system of hospital support, so far as this country is concerned, recognises the great underlying principle, that it is right of our own initiative, as a mere matter of the highest duty, to render some measure of personal service to the sick. This principle is the bedrock upon which the support of our great English hospitals rests. The voluntary system has produced the best results in regard to administration, management, progress and successful treatment within the hospitals, which the world has so far attained to. Hence the growing success of the Sunday and King Edward's Hospital Funds is due, to a widespread and true conviction, that the perpetuation of the voluntary system of hospital support is demanded in the interests of hospital efficiency, the adequate treatment of accidents and disease, and the well-being of the people at large. But, apart from the duty, there is the privilege which every thinking man and woman, and many young people too, enjoy, at Christmas time especially, when, in the midst of the joys of the family gathering, they give a little thought to the sick in the hospitals.

The best, and on the whole the truest idea of a voluntary hospital is, that it is a guest-house, where nearly everybody can secure the privilege of entertaining one guest at least. Some people complain that Christmas Day is so dreadfully dull, that it has outworn its welcome, and that the innocent amusements and frivolities associated with this season, are, in the present day, a weariness, rather than a joy, to the elders at any rate. Yet, in fact, unless a man has lost all sense of the joyousness of life in the struggle for existence, for pleasure, or for money, the children, with their innocent enjoyments and merry laughter, must prove inspiriting and helpful to him, in the highest and best sense. From a more selfish standpoint, it is essential to our health and well-being, as we grow older, that we should do everything to keep young, as long as possible. Nothing tends to aid us more in this direction than a hearty abandonment to the spirit of Christmas. Of course those who live to eat, forgetting that the true principle of life is merely to eat to live, must eventually reduce their thinking apparatus, as well as their internal organs, to a state of obesity, which will make them sometimes discontented with themselves, and always with the world at large. To such people Christmas should be a season, not of feasting but of fasting, for in no other way, in a physiological sense, can they hope to attune themselves to the spiritual side of their existence, which after all makes for good, for health, for life, for happiness, more than anything else in this world. There are people who realise this fact, and who prepare for Christmas by a period of relative abstinence, whereby their bodies gain tone, and their spirits attain a freshness and a vigour, to their lasting good and the immediate benefit of their friends.

The guest-house of us all, then, as every voluntary hospital, properly managed, undoubtedly is, in fact, must prove attractive to the thoughtful, as well as helpful to those who have eyes to see and hearts to feel. Instead of sitting about listlessly, or killing time as so many people strive to do with ill-concealed discontent, let everybody who can, make it their business, this year, to visit some hospital in the afternoon of Christmas day, where they will find the Christ spirit permeating the whole establishment, and making, even the sick, to rejoice and be glad. Most wards present something which is attractive and striking in the way of decoration, and every building contains guests who can be cheered and helped by each visitor, especially if he will try to realise something of the measure of the aggregate of all the suffering, represented by every form of disease and accident, to be met with in 600 beds in a great hospital in London. Go therefore and see, and learn, and act! Try whether the pleasure, and the actual benefit too, in the attainment of a spirit of thankfulness and joy, which may be derived from such a visit to the guest-house, do not prove helpful, not only for the day, but for the year which lies immediately ahead.

If it were possible to assemble, somewhere, the majority of the men and women of London, and to speak to them face to face on the subject of personal service in the days of health in the cause of the sick, we know from our experience of great audiences all over London, and in the home counties, that there would hardly be an appreciable minority, who would not experience, with the apprehension of the thought, higher aspirations, and the opening up for them of increased joys, the very existence of which very few realise.

Imagine that you are the patient, in your own bed in your own house, suffering from some grievous malady, pain-stricken, racked by disease, and anxious as to the result. For many years, it may be in God's mercy for a lifetime, illness has been a stranger to you, and so you feel keenly the affliction in the days of your sorrow. Lying there you begin to realise that health, and the power to do everything that you wish to do, as and when you please, have been accepted by you as your right, without any realisation of the blessing, or the source from which that blessing has come. And then the physician arrives, and he brings with him into the room an atmosphere of anxious endeavour to soothe your pain, to inspire you with hope, and to leave you cheered and comforted. And as you wonder how the doctor, with the care of all his patients, can bring into each room, or to each bedside, this personal atmosphere of charm, and hope, and confidence, you resolve, if you get better, to ask him the source of these individual gifts. What does the doctor say? That on the particular day, when you were most struck with the feeling of gratitude to him, for the help he rendered you, he will probably explain, that it was due mainly to the fact, that he had come direct from the hospital, the guest-house, where he had been working for two or three hours, doing his best for many poor souls, lying there as you were lying in your bed at home, but for the most part, suffering, more even than you were suffering, yourself.

He will probably ask you if you have ever been to a hospital; if you have ever thought of what London would be with its vast population of five million people, which is ever increasing and must increase with the empire, without the voluntary hospitals? Contrast with this, if you can, the measure of all the pleasure and enjoyment you have got out of your life, owing to the blessings of health which you have enjoyed, and ask yourself what have you done, in thought or in act, to aid the wayfaring man, who from no fault of his own, falls out by the wayside in the battle of life, struck down by illness, or overtaken by accident? You say you have never thought of these things, and the doctor will tell you, begin now, to think, and to act. Why? Because immediately you do, from a spirit and desire to render thanks for all the blessings you enjoy, by giving of yourself, as well as of your substance, in the days of health in the cause of the sick, you will begin gradually, though unconsciously, to experience a joy, which may find expression, as in the doctor's case, in a charm of manner and a grace of personal bearing, which will make your presence welcome wherever you may be. We ask sceptics, especially, to try the prescription, because if every sceptic were to do so, scepticism would die of inanition, before the world was much older.

For about forty years it has been our duty to advocate the cause of the hospitals. We suppose, in consequence, that any gift of writing which we may have possessed, must, almost necessarily, be losing its power, from the impossibility of writing freshly in such circumstances. The fault is ours, but it would not exist, if the will were strong enough to accomplish its desires, for the gospel of the guest-house, the invigorating and cheering effects of personal service in the cause of the sick, fill us still with joy, and make us desire, that this gospel shall reach, and become the spirit of action of, an ever increasing number of men and women, to their lasting happiness, and the immeasurable advancement of the nations of the world.

We publish, to-day, the needs of each of the hospitals of London. It will be observed that we have made no direct appeal of any kind this year to our readers. The reason is simple. All readers who realise what the guest-house should mean to them, apart altogether from what it means for the sick and suffering, will hasten, we are confident, to provide themselves with a guest, or guests, in some hospital this Christmas time.

### What Each Hospital Needs at the Moment.

In pursuance of our plan of taking the London Hospitals, and dealing with each hospital in order, we shall endeavour to bring out clearly what each is doing, what its present financial position is, and what are its most pressing requirements, which a benevolent public can advantageously further by a contribution in cash. We hope that the large number of wealthy people who have made it a practice to preserve the Christmas Appeal Number of The Hospital for reference throughout the year may be considerably increased this Christmas, and that all who use this issue, as a handy book of reference for the purposes of their charitable gifts, will find the plan pursued so helpful as to induce them to contribute even more liberally than they have heretofore done to the relief of sickness and accident in the metropolis of the Empire.

The Distribution Committee of the Hospital Sunday Fund has always had behind it the full confidence of the public, and we therefore quote the following figures from the particulars issued under its authority. It is commonly recognised that the great general hospitals are the backbone of our hospital system, and these striking figures in tabular form show (1) the number of beds, (2) the average number of beds daily occupied, and (3) the number of in-patients under treatment during 1903 in the greater hospitals of London—that is, those containing 150 beds and upwards.

| Name of Hospital. | No. of Beds. | Average No. of Beds daily Occupied. | No. of In-Patients Treated. |
|---|---|---|---|
| London Hospital ... ... | 816 | 676 | 12,460 |
| St. Bartholomew's Hospital ... | 674 | 536 | 7,264 |
| Guy's Hospital ... ... ... | 602 | 498 | 7,701 |
| St. Thomas's Hospital ... ... | 603 | 442 | 6,299 |
| Middlesex Hospital ... ... | 357 | 328 | 4,918 |
| St. George's Hospital ... ... | 351 | 269 | 4,198 |
| Hospital for Consumption, Brompton ... ... ... | 318 | 250 | 1,293 |
| St. Mary's Hospital ... ... | 281 | 255 | 4,023 |
| Dreadnought (Seamen's) Hospital ... ... ... ... | 269 | 235 | 2,501 |
| Hospital for Sick Children ... | 252 | 181 | 2,634 |
| King's College Hospital ... | 224 | 184 | 2,556 |
| Westminster Hospital ... ... | 213 | 179 | 2,681 |
| National Hospital for Paralysed | 195 | 185 | 1,066 |
| University College Hospital ... | 191 | 165 | 2,558 |
| Royal Free Hospital ... ... | 165 | 145 | 2,137 |
| City of London Hospital for Diseases of the Chest. ... | 164 | 124 | 848 |
| Great Northern Central Hospital | 159 | 144 | 2,081 |
| Royal National Hospital for Consumption ... ... | 154 | 146 | 795 |
| Charing Cross Hospital... ... | 150 | 107 | 1,566 |

The figures in the table show that the London Hospital passes many more in-patients through its beds than any other great hospital. Various explanations of the underlying cause of this have been given, but we are led to believe it to be mainly due to the large number of three-day cases treated at this hospital under the system there in force.

### Hospitals with 150 Beds and Upwards.

### GREAT NORTHERN CENTRAL HOSPITAL,

The work of this hospital is annually increasing, and has now grown so large that the premises are in many respects found inadequate to the demands. This is especially so in the in-patient department, and for some time past considerable difficulty has been found in accommodating the increasing domestic and nursing staff. The committee are determined not to allow the hospital to fall behind in the race for efficiency, and certain enlargements and extensions are already in progress. A new surgery and an additional operation theatre are now under construction, and a complete installation of medical electrical apparatus is designed. The great difficulty in the way is the want of funds, the utmost exertions being scarcely more than sufficient to make both ends meet. The hospital has to rely too much upon fluctuating sources of income such as legacies, and but for a certain amount of good fortune in regard to this item the institution would be in serious difficulties; hence the prospect of increasing expenditure attendant upon enlargement is one which requires some courage to face. The number of in-patients last year was 2,081, and of out-patients 25,998. Secretary, Mr. Lewis H. Glenton-Kerr, Holloway Road, N.

### GUY'S HOSPITAL.

All the wards in this great Institution are now open, more than 600 beds being available for patients. The cost of maintenance in 1903, apart from extraordinary expenditure, was £61,000. Towards this the hospital possesses an income, more or less assured, of some £35,000 per annum, and, in addition, has recently been in receipt of about £11,000 annually from King Edward's Hospital Fund for London, the Hospital Saturday and Sunday Funds, and subscribers, leaving a deficiency in ordinary income of about £15,000 per annum. Of the £180,000 appealed for in 1901, on renovation and building account, only £105,000 has been received to date, and for the balance the institution is in debt on temporary loans. The Governors earnestly recommend the claims of this historic and indispensable charity to be relieved from so embarrassed a financial condition and enabled to continue its great work, the second greatest of its kind in England, without curtailment.

### THE HOSPITAL FOR SICK CHILDREN.

This hospital is the oldest and largest Children's Hospital in the British Empire, and celebrated its Jubilee in 1901. It has 200 beds at Great Ormond Street, besides 50 beds at the Convalescent Branch, Highgate. Over 830,000 sick children have passed through this hospital in 50 years. There is a debt of £18,000, incurred in providing a house for the nurses and a garden for the children. £7,000 each year, or about £20 a day, is required to balance accounts, as the expenditure amounts to £19,000 a year, while the average income which can be relied upon is only £12,000. In the old days Great Ormond Street was the only Children's Hospital in the Empire, so that money poured in to meet daily expenditure. To-day there are 15 other Children's Hospitals in London alone, so that the "Mother of Children's Hospitals" is starved by the very success of the original movement started at Great Ormond Street in 1852. Probably no class of charity appeals more strongly to the sympathies than that which gives relief to suffering children, and it is therefore hoped that Great Ormond Street Hospital will receive the support it so much needs. Secretary, Mr. E. Stewart Johnson, Great Ormond Street, W.C.

### KING'S COLLEGE HOSPITAL.

As there is no room for expansion on the present site, and as there are other parts of London where the need of a general hospital is much greater, the authorities have made the very sensible decision to remove to a new district. The place chosen is Camberwell, where an anonymous donor has presented an area of 12 acres to the hospital. With this splendid site there should be little doubt that the new King's College Hospital when completed can be the most perfect and admirable institution of its kind in the country. Owing to the fact that Camberwell is a district which has hitherto been unprovided with a general hospital, and as it contains a very large number of the poorer classes among its residents, the sphere of usefulness of King's College Hospital is sure to be greatly increased by the move to fresh quarters In order to defray the cost of the removal the authorities have issued an appeal to the public for £300,000. Seeing that there is such a brilliant prospect before the institution it is highly probable that all the money needed will be forthcoming. The immediate requirements are £7,500 to pay off the loan from bankers and to provide for maintenance to the end of the year. Secretary, Captain H. S. Tunnard, Lincoln's Inn.

### THE LONDON HOSPITAL.

This hospital is situated in the East End, where its influence for good is not confined to the medical relief it affords, for the humanising and civilising influences which result to a patient, from a residence within its walls, are of immense value to the citizens. Its work improves the character of large numbers of the population throughout one of the poorest districts of London. This may also be said with truth in regard to other hospitals, but the influence in the case of the London is most patent and appreciable owing to the vast numbers handled every year. The annual income, including donations, subscriptions, legacies, etc., is not much over £60,000, and the cost of upkeep is £90,000. It would be a calamity to East London, if the work of this noble institution had to be curtailed. Its present circumstances justify special help, and even the £112,599 given by a generous public in answer to the Quinquennial Appeal of 1903 fell very far short of its immediate and pressing requirements, without furnishing a sufficiency for its future upkeep. Secretary, Mr. E. W. Morris.

### THE MIDDLESEX HOSPITAL.

The Middlesex, which is honoured with the patronage of his Majesty the King and her Majesty, Queen Alexandra, is one of the oldest established hospitals in the Metropolis; but has only a small endowment, and is chiefly maintained by voluntary contributions. There are 342 beds, all free, and during 1903 over 4,000 patients were received into the wards, and an average of 2,000 out-patients a week were under treatment. Important improvements and extensions of accommodation for patients and nurses, have been completed, the outlay entailed being £50,000. The Cancer Charity is a unique feature which distinguishes the Middlesex Hospital from other general hospitals. In accordance with the wishes Mr. Whitbread, the founder, expressed in 1792, research into the nature and cure of cancer has been continually carried on; but now, by means of methods of modern science, under a specially skilled director and staff of pathologists in the Cancer Research Laboratories, these investigations are being more completely pursued. The Convalescent Home at Clacton-on-Sea, contains 70 beds. The ordinary annual income requires augmenting by £12,000. Secretary-Superintendent, Mr. F. Clare Melhado.

### NATIONAL HOSPITAL FOR THE PARALYSED.

This Institution is an essential part of the hospital system in the metropolis, and in these days of hurry and strain every worker who has the means should make a point of giving something of his abundance to its support. In 1903 the hospital was honoured with the King's patronage and a Royal Charter of incorporation was granted. It relieved 6,366 out-patients and 1,004 in-patients last year. The fact that with 200 beds only 1,004 in-patients should have been admitted for treatment emphasises the special character and difficulty of the work done by this Institution for the public of all classes. During the last three years the accounts have shown a deficit of about £3,000 per annum, for which an urgent appeal is made. The Convalescent Home is to be closed at the end of the year for lack of funds. Annual subscriptions and donations are urgently needed for current expenses, as well as to carry out necessary extensions both in the in- and out-patient departments. We hope that financial assistance may be forthcoming without delay, as it most certainly will be if the wealthier classes realise what the work of this hospital means for everybody and how pressing are its claims upon them. Mr. G. Hamilton, Secretary, Queen Square, W.C.

### ST. GEORGE'S HOSPITAL.

The finances of this institution, considered in conjunction with the fact, that it is situated in immediate proximity to the wealthiest quarter of London, cannot fail to provide material for grave reflection. During the last three years the average gross annual income has been £34,195, and the average gross annual expenditure £45,602, showing a deficiency of nearly £12,000. Its pressing requirements are:—(1) a considerable expenditure upon the buildings and equipment of the hospital, and (2) the awakening of the wealthy residents around the hospital to the fact, that its income is too small for the work it annually does, to so large an extent in their especial interest. In 1902 an urgent appeal was made, but was very poorly responded to, less than £8,000 being received as the result. St. George's Hospital occupies the most conspicuous site of any London Hospital—a fact which, when taken in connection with the gradual falling off in the revenue from annual subscriptions, must be accepted as an indication, that the popular belief that it is desirable, for financial reasons, that the site of a big hospital should be in a leading thoroughfare, is largely erroneous. Mr. West is the Treasurer and Mr. C. L. Todd the Secretary.

### ST. MARY'S HOSPITAL.

This hospital ministers to the needs of the poor scattered over the large area included in Paddington and the neighbouring districts. Last year it relieved 43,513 out- and midwifery patients, in addition to 4,023 in patients. It has always enjoyed a wide and deserved popularity, but does not receive an adequate measure of support from those who reside within the area which it serves. When it is stated that Paddington, Kensington, and Marylebone, are the parishes whence the great majority of St. Mary's Hospital patients come, it cannot be urged, that the small proportion of well-to-do residents in those parishes is the reason for the small return which is made to the hospital, for its good work among their poor neighbours in the hour of sickness, for these parishes are among the richest in London. They contain thousands of opulent residents to whom a three guinea annual subscription to the hospital would be a mere trifle. Yet the total of annual subscriptions received by St. Mary's Hospital in the year was but £5,000, while its necessary annual expenditure was £30,000. The consequence is, it has to "kill the goose that lays the golden eggs," and spend all its legacies instead of investing them to create an income. Its financial position is, therefore, most precarious, and prompt and liberal help is necessary, if this great hospital is to be maintained in efficiency. Secretary, Mr. Thomas Ryan.

### SEAMEN'S HOSPITAL SOCIETY.

The varied work that is carried on in the establishments of the Seamen's Hospital Society is of the most interesting nature. Here the seamen are retained, not only during the severity of illness, but during convalescence. A very much needed addition has lately been made by the erection of a new out-patient department at the branch hospital in the docks. Although many of the patients are not properly objects of the charity in that they are not seamen, the relief of the hospital is open to them, and in this one department of the branch hospital, over 10,000 sick and injured receive assistance every year. The Seamen's Hospital Society urgently appeals to all who are free from the hardships and trials of those who live among the shipping in this East End centre of activity, to aid them in defraying the cost of succouring a most deserving class of the community.

Contributions may be sent to the Secretary, Mr. P. Michelli, at the *Dreadnought* Hospital, Greenwich, S.E.

### UNIVERSITY COLLEGE HOSPITAL.

THE buildings, newly erected through the munificence of the late Sir J. Blundell Maple, are approaching completion. It is expected that 86 additional beds and cots will on January 1st next be ready for occupation, making, with those at present open, a total of 277 beds. This further accommodation will enable the committee to admit many poor patients who cannot now be taken in for want of room. But the increased cost of maintenance which it is estimated will amount to £30,000 a year, is a subject of much anxiety to the committee. The hospital stands in close proximity to the densely populated district of Somers Town, which is inhabited by working men and women, of whom many are of the very poorest class. The number of beds at present available is 191, and the daily average number of in-patients during 1903 was 166, the total during the year being 2,558; 47,215 out-patients and casualties were treated, their attendances amounting to the large total of 165,253. To deal with this mass of suffering people, the ordinary expenditure, even with the most rigid economy, reached in 1903 the sum of £22,444 11s. 6d., whilst the reliable income from all sources was only £8,000, thus leaving £14,444 to be contributed by the benevolent public. This institution has never attracted the financial support which its position as the hospital of University College ought to have secured for it long ago. The pressing requirements this year are: (1) money to defray the cost of furnishing the new buildings; and (2) an additional income of £5,000 per annum at least. Beds or cots may be endowed and named in perpetuity as desired. Payments for the purpose may be made in one sum or by instalments. Secretary, Mr. Newton H. Nixon, Gower Street, W.C.

### THE BIRMINGHAM GENERAL HOSPITAL.

THIS hospital was founded in 1766, and the new building, which was erected and opened in 1897, has one of the most attractive and striking elevations of any hospital in the country. It is administered by a committee which is without doubt one of the most active and capable bodies charged with the management of a great hospital. It should have an immense interest for musical people of high and low degree, for the Birmingham Festival had its origin in, and owes its success to, the circumstance that it is held every three years in aid of this great medical charity. The hospital contains 346 beds; of these 289 were daily occupied during last year, when 5,366 in-patients and 64,926 out-patients were relieved. In 1903 the total ordinary income was £21,900, and the total expenditure £24,959, while the amount from the musical festival of £4,521 helped to reduce the deficit, which even now stands at £7,303. The most pressing requirement of this hospital is a considerable increase in the income to maintain the institution in full working order, and to meet the large and increasing demands made upon it year by year. House Governor, Mr. Howard J. Collins.

### ROYAL NATIONAL HOSPITAL FOR CONSUMPTION.

WITH the early arrival of severe weather the Board are receiving numerous poor applicants for admission. The advantages patients derive from this hospital include the open-air treatment in the mild climate of the Undercliff, and the provision of a separate spacious bedroom, facing south and overlooking the sea, with the best medical attendance and nursing. Although £3,000 is owing to the bankers, all the 155 rooms for patients are kept occupied. A subscription of £5 5s. yearly, or a donation of £52 10s. will constitute a governor, and any contributions will be gratefully acknowledged by the Secretary, 34 Craven Street, Charing Cross, W.C.

### HOSPITALS WITH UNDER 150 BEDS.

### CANCER HOSPITAL.

THIS hospital was founded in 1851 for the free treatment of those of the necessitous poor who are afflicted with cancer, tumours, or allied diseases. The nature of the disease makes it necessary to supply both food and dressings in great quantity and of high quality. The annual expenditure amounts to about £12,000, to meet which there are annual subscriptions of £2,000 and dividends £4,000. The balance has to be made up by donations, etc, and generally it means selling capital to the amount of about £3,000 or £4,000. Recently the pathological department has been much enlarged, a photographic section being added, an electrical department organised, and the treatment by radium undertaken. A new nurses' home has also been built and formally opened by Lady Ludlow. These necessary improvements have greatly added to the efficiency of the hospital and also to the cost of maintenance. Secretary, Mr. F. W. Howell, Fulham Road, S.W.

### EAST LONDON HOSPITAL FOR CHILDREN, SHADWELL, E.

EVERY time we enter the East London Hospital for Children we are more and more impressed with the quality of the work it is doing for East London To pass out from the gloom and squalor of the surrounding streets into the wards of this hospital is a surprisingly encouraging and pleasant experience. Of its 135 beds, 118 were daily occupied last year. It relieved 1,723 in-patients at Shadwell, and 375 at the seaside branch at Bognor, in addition to 33,065

out-patients. But so inadequately has this important work been supported, that instead of paying off the £1,630 owing at the close of last year, it is in still greater want this Christmas, for the hospital has been compelled to borrow yet another £1,000 from its bankers. It is moreover feared that before the end of the year the total indebtedness will have increased to £4,000 unless material help is forthcoming. The hospital had never, until last year, been driven to borrow money, and it would indeed be deplorable, if its invaluable work, in one of the poorest quarters of London, should now be impeded, through inability to rid itself of this burden. Contributions should be forwarded to the Secretary, Mr. Thomas Hayes.

### LONDON FEVER HOSPITAL.

Many people subscribe to the hospital to secure a right of admission ; some from pure benevolence, and others in recognition of benefits received from the hospital either for themselves or for members of their households. All alike are entitled to these privileges ; but, happily, comparatively few are obliged to use them in each year. The money thus saved enables the Committee to offer treatment to those of the general public who are attacked by the diseases named, and who are able to pay about a fourth of their cost to the hospital. Over 1,500 such persons have been received in the last three years. They are people who decline to cast themselves upon the rates in infectious sickness ; who are able to pay a part at least of the cost of their illness, and who if they remained for treatment in their own homes, could hardly fail to be a source of danger to those about them. There are private rooms for patients who can pay the cost of their illness. During the last 100 years over 82,000 sufferers have been received. Who can estimate the number of lives that have been saved in all classes of society by a century of such work? Every household should be interested in it, for the diseases named are no respecters of persons. The hospital receives no aid from the rates, and it stands in danger of languishing for want of adequate help. Secretary, Major W. Christie, Liverpool Road, N.

### THE LONDON HOMŒOPATHIC HOSPITAL.

The urgent need of this hospital is an increase of annual subscriptions. The sum needed for each day's maintenance is 30 guineas, while the income for each day is under 21 guineas. It will therefore be seen that a considerable extension of the list of annual subscribers is required. It is an exceedingly active hospital, comprising not only general medicine, but various special departments and an electrical department for the treatment of lupus and other diseases. The Homœopathic Hospital has many claims upon public support. It has been described as one of the most beautiful hospitals in London, and it is certainly one of the most economically conducted. Last year 1,145 in-patients were relieved, and the out-patient consultations numbered 43,889. The hospital is open to inspection every weekday between 10 and 4, when visitors will receive a hearty welcome. Cheques should be addressed to the secretary at the hospital, Great Ormond Street, W.C.

### LONDON LOCK HOSPITAL AND RESCUE HOME.

Help is very much required for this valuable institution, which is the only one of the kind in this or any other country, with the hospital and home conjoined. There has been a continual flow of patients to the Hospital, some of whom have found their way to the Home and other homes, in many cases with very good results. It is the desire of all connected with the Hospital and Home to do the utmost in their power to succour those who are in want of a helping hand. Secretary, Mr. A. W. Cruickshank. Offices, Harrow Road, W.

### METROPOLITAN HOSPITAL.

This institution is situated in one of the poorest and most crowded, and therefore most suitable districts of London. It deserves a much larger measure of support than it has heretofore succeeded in attracting. Of its 111 beds 97 were daily occupied during last year, and it relieved 1,270 in-patients and 34,111 out-patients. During the last three years the average gross annual income was £13,985, and the average gross annual expenditure £13,776. Some £20,000 are urgently required for a nurses' home in order to free for the reception of patients the wards now converted into cubicles. The secretary, Mr. Charles H. Byers, Kingsland Road, N.E., has for many years laboured assiduously and successfully to extend the resources of this important institution, which in the past has been far too little known to wealthy contributors to hospitals—a disadvantage which we hope will speedily disappear.

### THE MOUNT VERNON HOSPITAL FOR CONSUMPTION AND DISEASES OF THE CHEST.

Particular attention is drawn to the urgent need of support in which this hospital stands to enable it to extend its accommodation from 130 to 250 beds, and thus meet the growth in the demands upon it. The duties of the medical officers are of an exceptionally difficult and distressing character, for they are compelled to daily refuse applicants eligible for indoor treatment, whose lives might be prolonged by admission to the hospital. But it is still more painful to relate that for those who are approved—and nearly 300 are waiting their turn to be admitted—there remains the long and weary wait of at least 15 or 20 weeks, during which time the disease which has attacked them pursues its course with such deadly effect, that many whose cases were hopeful when approved, may become hopelessly ill before they can be received. It is to remedy these unsatisfactory conditions that help is required. The £15,000 immediately needed ought surely to be forthcoming, especially as the hospital is doing such good work in the treatment of those suffering from consumption and other diseases of the chest. Secretary, Mr. W. J. Morton, 7 Fitzroy Square, W.

### NORTH-EASTERN HOSPITAL FOR CHILDREN.

This hospital is now in a position of exceptional need. The expenditure was increased from £6,500 to £11,000 a year by the opening of the new wards which doubled the number of beds, and as the income has been stationary a debt of £4,000 has been incurred in the maintenance of the increased work. There is besides, a debt of £9,000 on the new building, and the two together constitute as heavy a burden as the committee consider the hospital ought to be called upon to bear. They have accordingly decided to close 57 beds at the end of this month unless sufficient assistance is forthcoming in the meantime. The 114 beds in the enlarged hospital are in constant use, and to withdraw half of them would be a disaster to the poor of the neighbouring districts. The whole neighbourhood is aroused on the subject and is doing everything possible to aid the committee to ward off the threatened calamity. The clergy have formed an Emergency Committee amongst themselves to assist in raising the £4,000 required by the end of the month. The accident and emergency cases brought to this hospital number 250 per week in addition to 1,200 out-patients, and the 114 beds are always full. Secretary, Mr. T. Glenton-Kerr, Hackney.

### ROYAL LONDON OPHTHALMIC HOSPITAL (MOORFIELDS EYE HOSPITAL).

In this hundredth year of the hospital's existence the committee specially appeal for new annual subscriptions and donations. To those who may not know the work of this hospital, the committee briefly state that: It is the oldest and largest eye hospital in the kingdom: open free to the poor suffering from disease of or injury to the eye. The hospital relieves over 100 in-patients, and over 400 out-patients every day. Contributions should be sent to the Secretary, Mr. Robert J. Bland, City Road, E.C.

### WEST LONDON HOSPITAL.

The annual income from endowments barely exceeds £300, while the annual expenditure upon present work (over 2,000 in-patients and 35,000 out-patients) is about £12,000. An exceptional and heavy expenditure of about £6,000 had recently to be undertaken, towards which the King's Hospital Fund contributed £2,000, leaving £4,000 to be otherwise provided. The hospital owes its bankers £10,850. The hospital is very urgently in need of help. Secretary, Mr. R. J. Gilbert, Hammersmith.

### Hospitals with Under 100 Beds.

### THE CHELSEA HOSPITAL FOR WOMEN.

This hospital, which has 50 beds, was founded in 1871 for the reception and treatment of respectable poor women and gentlewomen in reduced circumstances, suffering from the distressing diseases to which their sex is peculiarly liable. The council appeal for donations and new annual subscriptions (their only reliable source of income) in order to defray the cost of alterations carried out to give the patients the benefit of the latest medical science, without incurring a heavy debt, and to pay off a mortgage of £4,000 on the hospital. There is a convalescent home at St. Leonards-on-Sea, which contains 22 beds, and is open to others than those who have passed through the hospital. Secretary Mr. Herbert H. Jennings, Fulham Road, S.W.

### THE HOSPITAL FOR WOMEN.

This is one of the most comfortable hospitals in London from the patients' point of view. There are 60 beds, all in constant use. The committee very earnestly appeal for additional annual subscriptions and donations for their maintenance. Secretary, Mr. David Cannon, Soho Square, W.

### NORTH-WEST LONDON HOSPITAL.

This is the only institution of the kind in the north-west district and it was founded in 1878. It has 50 beds, and last year there were 615 in-patients and 44,465 attendances of out-patients. It has great claims upon all thoughful persons from the circumstance that Mr. George Herring, who is probably the most beneficent of the regular contributors to metropolitan hospitals, takes an abiding interest in its upkeep and maintenance. There is great need for a large sum to enable the committee to get out plans and erect a modern hospital, so that the inhabitants of the north-west district of the metropolis may be adequately supplied with hospital accommodation. The present work, entails a yearly expenditure of £4,500, towards which the income from annual subscriptions at present yields less than £700. The hospital has been generously helped by King Edward's Hospital Fund, and the Hospital Sunday and Saturday Funds. We believe if this deserving hospital were more widely known it would not be so crippled for want of funds. Secretary, Mr. A. Craske, Kentish Town, N.W.

### POPLAR HOSPITAL FOR ACCIDENTS.

Situated amongst a teeming population of poor hard-working people in a district which may be called the "workshop" as well as the "port" of London, this hospital is doing an excellent work. The demands on the institution have of late years greatly increased, and consequently further subscriptions and donations are very necessary. Secretary, Lieut.-Colonel Feneran, Blackwall, E.

### QUEEN CHARLOTTE'S LYING-IN HOSPITAL.

This hospital received 1,444 patients into its wards last year, and, in addition, attended 1,577 patients at their own homes. Although upwards of £25,000 have been spent during the past few years in enlarging and modernising the hospital and in building a nurses' home, the demands on the accommodation of the charity have been so great that the committee have had to acquire additional premises adjoining the nurses' home for an enlargement of the home. The cost of these enlargements will exceed £5,000, towards which contributions are earnestly solicited. Funds for general maintenance are also greatly needed, for, while the expenditure amounts to nearly £6,000, the reliable income is but little more than £2,000. Donations may be sent to, the Secretary, Mr. A. Watts, Marylebone Road N.W.

### Hospitals with Less than 50 Beds.

### CITY OF LONDON LYING-IN HOSPITAL.

The construction of a tube railway in close proximity to this hospital (which had been erected over 130 years) caused such serious damage to the foundations and structure that the committee, acting upon the advice of experts, have decided to rebuild the old portion of the hospital, which was considered to be in an unsafe condition. This, it is estimated, will cost £25,000. The committee appeal for funds towards this object, and the Lord Mayor has consented to preside at a festival dinner in May next (the first since 1830). In order that in-patients may not be deprived of the benefits of this hospital during rebuilding, temporary premises in Old Street have been secured, entailing an additional expenditure of £1,000 a year. It therefore will be seen that this, one of the oldest lying-in hospitals in Great Britain, is in the greatest need of liberal assistance. Contributions will be welcomed by Mr. R. A. Owthwaite, the Secretary, City Road, E.C.

### HAMPSTEAD GENERAL HOSPITAL.

A new hospital is in course of erection, and further assistance to the extent of £12,000 is required. The old buildings contain 35 beds, and in the past eleven months 414 in-patients were relieved, while there were 7,175 attendances of casualty and out-patients, included in which are 182 accident and emergency cases, and 560 minor accident cases. The large number of serious accident cases treated, proves how well situated this hospital is. The value of this hospital has been recognised by King Edward's Hospital Fund, which has helped substantially towards the Building Fund. There is an average annual deficit of £550. The Council earnestly plead for help both towards the Building Fund and to meet current expenses. Secretary, Mr. George Watts, Parliament Hill, N.W.

### HOSPITAL FOR EPILEPSY AND PARALYSIS.

This hospital has been rebuilt upon a new site in Maida Vale and has been opened since the end of 1903. We have far too few beds for the accommodation of patients suffering from paralysis and epilepsy and the numerous diseases of the nervous system, and a contribution towards the large sum required each year to maintain this institution would be well laid out. Mr. H. Howgrave Graham, the secretary, has devoted many years of his life to the difficult task of maintaining this hospital, and we hope that many charitable people will visit it and identify themselves with its work. It needs both friends and funds.

### MILLER HOSPITAL AND ROYAL KENT DISPENSARY.

An exceptional opportunity for extension has just presented itself, the committee having received nearly £2,700 from the fund raised in 1902 by the mayor and the committee nominated by the Borough of Greenwich Council, to commemorate the Coronation of his Majesty King Edward VII. The scheme for the enlargement of the hospital comprises the erection of a new wing with 20 beds, better accommodation for out-patients and casualties, a suitably fitted ophthalmic department, and extra accommodation for nurses. The new wing and the other improvements named, including the cost of the two houses, will amount to £10,000, and towards this sum the committee have in hand or promised about £5,000, including a liberal grant of £1,000 from King Edward's Hospital Fund. If the further £5,000 can be raised, the whole of the scheme for enlargement and improvement can be carried out. The committee earnestly appeal for help towards this object. Secretary, Mr. James Marks, Greenwich.

### PADDINGTON GREEN CHILDREN'S HOSPITAL.

This hospital was established in 1883 and is supported entirely by voluntary contributions. It is quite free, without letter of recommendation, to the sick children of the poor, and provides 46 cots. In 1903, 654 in-patients were admitted, while there were 15,727 new out-patients and 1,079 casualties and accidents, the total attendances of out-patients being 50,555. The hospital has a convalescent home, with accommodation for 16 children, at "Fair View," Slough, for patients who have passed through the wards. One hundred and thirty-nine children were admitted in 1903. Funds are greatly needed for maintenance purposes and also to pay off a debt of £2,500. Visitors are invited to inspect the hospital, or home, any day (except Sunday) between 2 and 4 p.m. Treasurer, George Hanbury, Esq., 28 Prince's Gate, S.W.

### ST. SAVIOUR'S HOSPITAL.

This hospital has now been for 12 years under its present management, and on an average 100 patients have been treated annually. It is intended exclusively for that special class of patients—ladies of limited means—to whom the necessary publicity of a general hospital, and the heavy expense of a nursing home, are alike insupportable. It is one of a very few hospitals in London which makes any attempt to supply the want, acknowledged on all sides to exist, of adequate hospital provision for that large class of patients who desire privacy, and can pay some small sum towards their treatment. Needless to say the patients' payments at St. Saviour's do not meet even half the expenses incurred, and lady readers, who have a sufficiency, may well remember this, and realise that the work this hospital is doing is for the benefit of their less fortunate sisters. Communications should be addressed to the Honorary Secretary, Osnaburgh Street, N.W.

### SAMARITAN FREE HOSPITAL FOR WOMEN.

This hospital is entirely free, and is dependent upon the charitable public for £6,000 per annum to meet the cost of its maintenance. Funds are wanted at once to meet outstanding liabilities. The committee appeal for bequests and donations towards a new endowment fund; £1,000 will endow and name a bed. £15,000 is still required to build a new outdoor department, improve the accommodation for nurses, and build a new operating theatre. Secretary, Mr. W. G. King, Marylebone Road, N.W.

### WEST END HOSPITAL FOR DISEASES OF THE NERVOUS SYSTEM.

This hospital is devoted to the good work of giving relief to paralysed and nervous children, and applications for treatment increase every year. The institution has no endowment or funded property whatever, and is entirely dependent upon the subscriptions and donations of the generous public to carry on its daily work. It is in very pressing need of a convalescent home by the sea, where patients could be sent for a time after their discharge from the hospital, and the committee are striving so raise the necessary funds to carry out this scheme. Contributions towards this object and to the expenses of maintenance should be sent to the Secretary, Mr. Alfred J. Wise, at the Hospital, 73 Welbeck Street, W.

### Some Poor Folk and the Poets.

Shakespeare's advice to ordinary mortals in the conduct of their ordinary affairs applies with special force to those who take little or no part in the field of charity. Here it is:—

> Neither a borrower nor a lender be,
> For loan oft loses both itself and friend;
> And borrowing dulls the edge of husbandry.

Shakespeare's allusion to husbandry reminds us of another catch which may be worth quoting here:—"He who goeth a-borrowing, goeth a sorrowing." In matters of charity—it almost amounts to a national fault—too many people have the habit of doing their alms vicariously. Some friend tells them of, say, a widow and her children where the bread-winner has suddenly died. In such a case a large number of worthy people, instead of putting their hands in their own pockets or taking personal trouble to ascertain if there is something they can do directly to help the family, express sympathy, and suggest an orphanage for the children, to which, as a rule, they do not subscribe themselves. If they are unusually energetic they may even offer to write and ask a few of their friends to vote for the child at an ensuing election. Having done this they are contentedly conscious of their great kindness to friends and orphans alike. Yet there is no real charity in this vicariousness, for the course indicated, means in practice, that the individual is content to borrow charity from one friend without cost or obligation, in order to bestow it on an object in which another friend is interested. But such individuals say they feel inspirited, it may be, by their generous action in taking so much trouble, though they have not the smallest intention of repaying the loan or discharging the liability which they have incurred. To mean people there must surely come a day of reckoning, unless they are so callous and seared by contact with the world and its affairs, that they pass out of existence without any realisation of the spiritual side of their nature, or its responsibilities.

We will commence, then, our request for attention to the claims of the General Charities, by expressing a hope that none of our readers at any rate will be content to borrow where charity is concerned. We believe the day is not far distant, when the English people will substitute for the orphanage, a system of individual care, by placing all orphans in families, and taking care that those who deal with each case shall give enough time to see that the child's welfare is well provided for in the adopted home.

The General Charities! How little meaning, we fear, is conveyed by the words. Yet the relief of every form of chronic suffering known to man is here included—the oral instruction of the deaf and dumb, provision for the blind, for the orphans of both sexes and all classes who may need it, for the relief of the shipwrecked fishermen and sailors, for the convalescent care of those recovering from serious illness, for the succour of distressed ladies, dinners for the poorest children in public schools, and a home for convalescents after infectious disease. Such are some of the general charities, and when it is recognised that in all cases where children are concerned the provision made includes a thorough education and technical training, with an attempt to start them well in life, there will surely be found, amongst them all, some one object, which must successfully appeal to every person with a mind intelligent enough to realise his responsibility.

Let us next endeavour to bring out tersely but clearly, why, from the very circumstances of many of those whose cause we plead, the more fortunate and happy of our race should welcome the opportunity to lend a helping hand. Let us take the case of the blind. Has the reader ever visited a blind asylum or school, to see what is done there, and the effect of the work upon both children and adults? If not, seeing that blind institutions are situated up and down the country, and in many parts of a great centre like London, we would ask the reader to devote an afternoon to such a visit, and we are confident he will repeat it from choice. For knowledge creates a sense of hunger, and hunger promotes endeavour, and endeavour excites attachment to causes and individuals, which may one day add to the joy of living. It is inconceivable that any person blessed with eyesight can fail to sympathise with the blind :—

O! dark, dark, dark, amid the blaze of noon!
Irrevocably dark! total eclipse, without one hope of day.

It must be terrible, indeed, to be born blind, but, to quote Shakespeare once more, there is something even worse, for "he that is stricken blind, cannot forget the precious treasure of his eyesight lost." This is an age of insurance, and we are availing ourselves of its advantages more and more. Give something, then, oh people of strong vision, in the full enjoyment of your sight, during your halcyon days, to the cause of the blind.

Cynics have said that the world would be durable as a place of residence if the human animal were dumb. In truth, however, the power of speech is one of the most blessed of gifts; it promotes progress; it adds immeasurably to the joys of living; it attracts and binds together kindred souls; it moves peoples to deeds of heroism and to acts of nobility; it raises men from the level of the animal kingdom to the highest regions which the mind can penetrate; and without it the world would make little progress, and men and women might soon sink back to mere animal existence. All this is little apprehended by the majority of the race, and few indeed spare a thought for the dumb who are frequently deaf as well. Yet they exist, and move, and have their being amongst us. Their condition and lot should excite our pity, and we should all try to do something, at any rate, to make their lives as useful and endurable as possible. It is of course true that the deaf and dumb owe their infirmity to God's unseen providence, but it is the privilege of the hale to shield them from man's cruelty, by providing for their necessities to the fullest extent which money can secure. On their own merits modest men are dumb, and properly so, but let us all be eloquent on the merits of the deaf and dumb, for

A beggar that is dumb, you know,
May challenge deeper pity.

The fate of the orphan is one which appeals directly to the heart of mothers and fathers, and even to young people who are just entering upon their lives. Indeed, the very helplessness of these unfortunates, smitten sometimes at their birth by the cruel hand of fate, casts upon everybody, irrespective of religious belief or sentiment, the duty of caring for the orphan. In times like the present, when the whole world is conscience-stirred by the horrors which war is inflicting upon hundreds of thousands of innocent people, directly on the battlefield, and indirectly in the homes of the slaughtered men who have fallen in Manchuria, few words should be needed in advocacy of the claims of the orphan. Those in high places, who are immediately responsible for wars, and the general who sacrifices the lives of his men without warrant, are credited by Shakespeare with a haunting vision which we may here record :—

Some undone widow sits upon mine arm,
And takes away the use of it; and my sword,
Glued to my scabbard with wronged orphans' tears,
Will not be drawn.

These words have a meaning which all can apply, for war in these days is not confined to battlefields, but is being continuously waged in the struggle for existence, especially in large cities, all the world over. Do we all do our utmost always to ease the struggle for the weaker brethren?

Where would London be without its sailors and mariners? And who has not a warm place in his heart for Jack? At this season, too, perils of the sea are constantly brought to our notice in the papers, and the sympathy with all who have to face them is keen and real.

But hark! what shriek of death comes in the gale,
And in the distant ray what glimmering sail
Bends to the storm? Now sinks the note of fear!
Ah! wretched mariners! no more shall day
Unclose his cheering eye to light ye on your way!

And what of those who are left behind? Are not they in the custody of the people of these islands? So too are the men who escape with their lives, but lose their bodily possessions. Surely the general charities which provide for seamen and mariners will have no cause to be dissatisfied with the response of the public to their claims at this season.

Finally, let every reader endeavour to see in his mind's eye this procession of blind, deaf and dumb, shipwrecked, distressed, disease haunted, and orphaned army, which the general charities aim at providing for. The particulars given by each institution speak for themselves. Let the motto of each reader be, "To all according to their needs, from each according to his means." And to the sceptic and indifferent we would say, we beg of you good friends—

"At Christmas time, in pleasant mood,
Come, try the luxury of doing good."

### The General Charities.

### ASSOCIATION FOR THE ORAL INSTRUCTION OF THE DEAF AND DUMB.

This association is entitled to the credit of being the first to publicly introduce into the United Kingdom the pure oral system for teaching deaf and dumb children to speak *viva voce* and to understand the spoken words of others by lip reading. The expenses, which are very heavy, are met by voluntary contributions and fees, which fall short of the amount imperatively needed to the extent of about £600 per annum. Director Mr. William Van Praagh, 11 Fitzroy Square, W.

### THE CHILDREN'S HOME.

There are at present 1,500 children in the ten branches of the Children's Home. A new branch, the gift of Miss Fowler, of Liverpool, is in course of erection at Frodsham. There is also an Emigration Home for boys, in Canada. A special feature is the home life of the children, who are gathered into small communities, each with its own family circle, and its own interests, under the care of a "Sister-of-the-Children." Unfortunately the need for funds is urgent and extreme. £16 will support a healthy child for one year, and £30 will provide for a cot in one of the hospitals or the Cripples' Home for a like period. Donors of £50 are entitled to name a cot. All communications and remittances should be sent to Rev. Arthur E. Gregory, D.D., Bonner Road, N.E.

### FIELD LANE RAGGED SCHOOLS AND REFUGES.

The president and treasurer of this institution invite special attention to its services and necessities. Some idea will be conveyed of the operations in which the institution is engaged, which involve large expenses and great personal exertions, when it is stated, that for the year ending March 31st last, 275 persons were provided with employment, 748 men and women were sheltered in the Refuges, 159 boys were maintained in Industrial Home, and 273 children were sent to country holiday homes. The attendances at the Crêche, Ragged Church, Adult Mission Services, Bible Ragged Schools and Classes, Band of Hope, Gospel Temperance Society, and Mothers' Meetings amounted to over 146,000, while 92 bags of linen were lent from the Maternal Society, and distributions of bread and broken food were made to over 40,000 persons. The committee are burdened with a debt of £4,000 to their bankers—a deficiency which has accumulated during the past few years. Every department is fitted to meet the temporal and spiritual needs of the destitute poor of this vast metropolis. To maintain the work at least £7,500 a year is required. An average of £2,500 is received from the Home Office and the London County Council towards the expenses of the Certified Industrial School, leaving £5,000 to be made up by voluntary contributions. The necessity for the continuance of the Institution is great, and a very earnest appeal is therefore made for help in its present urgent need. President, The Earl of Aberdeen; Treasurer, W. A. Bevan, Esq. Contributions should be forwarded to the Secretary, Vine Street, Clerkenwell Road, E.C.

### FREE CONVALESCENT HOMES.

To take up cases amongst the sick poor, where the hospitals and dispensaries leave them, to complete cures which physicians and surgeons have so well begun, is the commendable function of the charity. The Home, which was the first of the kind established in this country, commenced its beneficent work 64 years ago. Its operations have been steadily extended until now it maintains four homes, containing 541 beds, to which nearly 7,000 patients are admitted every year, for a period of three weeks, entirely free of charge. A new Home at Little Common, Bexhill, for men only, which will accommodate at first 71 patients, is nearing completion. Since 1840 no fewer than 158,794 adults and 37,440 children have been received, the greater number of whom were fully restored to health and strength. In spite of strict economy, the cost of maintaining the four homes is about £13,000 a year, nearly all of which has to be obtained from voluntary sources, and a very earnest appeal is made for additional annual subscriptions and donations. The Secretary, Mr. Alex. Hayes, will gratefully receive subscriptions and donations at the office, 32 Sackville Street, W.

### THE IRISH DISTRESSED LADIES FUND.

The chairman of the London and Irish Executive Committees of the Irish Distressed Ladies Fund appeal to the public through our columns on its behalf. They speak from personal experience of the good work that has been done by the London and Dublin Committees in assisting ladies who have been reduced, through no fault of their own, but in consequence of agrarian troubles. The receipts to October 31 were £1,619 14s. 3d., and the expenditure during that period has been £2,445 6s. 11d., consequently it has been necessary to draw upon the moderate reserve to meet urgent demands. There are 67 pensioners on the books, chiefly living in Ireland, and 96 ladies are helped to earn their living through the work depôts in London and in Dublin; £1,091 has been spent in pensions, £428 10s. in special grants and help in cases of illness, and £169 13s. 4d. towards the education of children. While being well aware of the numerous calls which are made upon the benevolent public at this season of the year, it is hoped that those who have favoured this good work in the past with help will continue it on this occasion, and that others may be induced to assist materially too. Secretary, General W. M. Lees, 411 Oxford Street, W.

### THE LONDON ORPHAN ASYLUM.

The Board of Managers are urgently appealing for increased support in aid of this important charity, both towards its current expenditure and for its permanent benefit. Orphans from every part of the British Empire are eligible. Nearly 500 are in the school. Seventy candidates are seeking admission, and the managers have determined to admit, as many as possible, in January, relying upon the help of the public to support them in undertaking the additional liability. Over 6,570 orphans have been already benefited. The benevolent may unhesitatingly support this charity, as its funds are administered with every regard to economy, which is consistent with efficiency. £13,400, however, is required from voluntary sources each year. In the words of the founder—"The widow and the orphan have an undisputed, perhaps an unrivalled, claim to our benevolence. They arrest, as by a common feeling, the sympathies of all. And if our wealth, our influence, and our talents are thus employed, while the season of action continues, in circumstances of distress and seasons of suffering and incapacity, which alike affect the whole of our race, we may delight ourselves with the reflection of a venerable patriarch—'When the ear heard me then it blessed me; and when the eye saw me then it gave witness to me; because I delivered the poor that cried and the fatherless, and him that had none to help him. The blessing of him that was ready to perish came upon me, and I caused the widow's heart to sing for joy.'" Secretary, Mr. Henry C. Armiger, 21 Great St. Helen's, E.C.

### THE MARY WARDELL CONVALESCENT HOME FOR SCARLET FEVER.

It is very desirable that there should be a sum raised to be invested so as to produce a small endowment sufficient to meet at least the greater part of the salaries and wages of the nursing and domestic staff of this home. The nature of the illness makes the numbers of patients very variable, and though their payments do not cover their cost, they go a good way towards it in seasons when the home is full. But when there is little scarlet fever the home has to be kept up in full working order, and is then entirely dependent on subscriptions and donations, and there is much difficulty in carrying it on. Subscriptions and donations may be sent to Messrs. Barclay and Company, and to Miss Mary Wardell, hon. secretary, Stanmore, Middlesex.

### ORPHAN WORKING SCHOOL.

This is a national and undenominational institution, and maintains 500 children, varying in age from infancy to fourteen or (in special cases) fifteen years. It is greatly in want of funds at the present time, and an appeal is made specially for new annual subscriptions, the normal income of the charity being more than £2,000 below what is needed to meet the expenditure. Secretary, Mr. Alexander Grant, 73 Cheapside, E.C.

### ROYAL ALFRED AGED MERCHANT SEAMEN'S INSTITUTION.

At the present time this institution is maintaining 100 old seamen at the comfortable home at Belvedere, and sending to 450 others a pension of £1 per month at their own homes, throughout the United Kingdom. Although, during the year 90 old tars were admitted to the benefits of the institution, and 100 more will be elected in January next, the authorities cannot keep pace with the ever-increasing list of urgent cases, that are daily coming before them. It is, therefore, hoped that the necessary help will be forthcoming, to enable this institution to meet the demands made upon it by decayed seamen, to whom this country owes so much. Secretary, Mr. J. Bailey Walker, 58 Fenchurch Street, E.C.

### BOURNEMOUTH NATIONAL SANATORIUM FOR CONSUMPTION AND DISEASES OF THE CHEST ("OPEN-AIR" TREATMENT.)

During 1903 patients were received from 31 different counties in England, besides others from Wales and Ireland. The marked success which has attended the hygienic or "open-air" treatment of consumption, together with the favourable situation and climate in which the sanatorium stands, lead many hundreds of poor sufferers to seek admission each year, and applications have increased so largely that it has been necessary recently to further enlarge the institution. This extension will be shortly ready for opening, when additional accommodation will be provided for about 80 more patients yearly. The outlay will amount to £2,700, of which less than £1,900 has been subscribed in three years, leaving over £800 yet to be obtained to pay the builders. A further £200 is required for equipment. There has been a great falling-off in voluntary contributions this year for ordinary purposes, and £500 is urgently required to meet current expenses. The total sum needed before the end of the year to clear the sanatorium from debt is £1,500. In addition to the present financial needs, the ordinary annual expenditure will be permanently increased in order to maintain the additional patients for whom extra accommodation is being provided. For this purpose new annual subscriptions to the amount of £500 are necessary, without which it will be impossible to keep the whole of the Sanatorium open. The committee, therefore, most earnestly plead for liberal assistance. Treasurer, G. J. Fenwick, Esq.; Secretary. Mr. A. G. A. Major.

### THE ROYAL ORPHANAGE, WOLVERHAMPTON.

Special attention is called to the work this institution is doing for London. There are now 15 children in the Royal Orphanage from London and its immediate neighbourhood, representing an annual expenditure of about £300. The total amount of subscriptions, etc., received to meet this large outlay is £32 10s., and the board of managers therefore feel that they have a claim to more adequate support from Londoners. This institution affords the advantages of home and education to fatherless children of either sex who are destitute of the means of support, and who are retained in the institution till 15 years of age. There are now 320 children in the establishment, which is capable of receiving a much larger number when sufficient funds are obtained for their support. The institution has accommodation for 400 children. The subscribers have the power of nominating candidates, and the right of voting at all elections, according to the amount of their subscriptions. An earnest appeal is made for additional support that the usefulness of the charity may be extended Secretary, Mr. Walter Hamblett.

### Great Northern Central Hospital,

### HOLLOWAY.

**President—H.R.H. THE PRINCE OF WALES, K.G.**

CHAIRMAN—SIR JOHN DICKSON-POYNDER, BART., M.P.

In the Midst of North London's Poorest Population.

**27,000 Patients Relieved Annually.**

This Hospital **REQUIRES £14,000 A YEAR** in order to keep its Wards open, and has only **£4,000** in reliable income.

**DONATIONS, SUBSCRIPTIONS, and LEGACIES** to meet the Heavy Annual Deficiency are Earnestly Requested.

LEWIS H. GLENTON-KERR, *Secretary.*

### QUEEN CHARLOTTE'S LYING-IN HOSPITAL

### MARYLEBONE ROAD, LONDON, N.W.

FOUNDED 1752. INCORPORATED BY ROYAL CHARTER, 1885.

**UNENDOWED. SUPPORTED BY VOLUNTARY CONTRIBUTIONS.**

*Patron*—HER MAJESTY QUEEN ALEXANDRA.
*Vice-Patron*—H.R.H. THE PRINCESS OF WALES.
*President*—THE RIGHT HON. THE VISCOUNT PORTMAN.
*Treasurer*—ALFRED C. DE ROTHSCHILD, ESQ., C.V.O.

### OBJECTS OF THE CHARITY.

1.—The Delivery of Married Women, both in the Hospital and at their own homes.
2.—The Delivery of deserving Single Women, in the Hospital, *with their first child only.*
3.—The Training of Medical Pupils, Midwives, and Monthly Nurses.

Since the foundation of the Hospital, 100,000 POOR WOMEN HAVE BEEN RELIEVED. Last year 1,444 Patients were received into the Hospital, and 1,577 were attended at their own homes.

THE ANNUAL EXPENDITURE of the Charity amounts to nearly **£6,000,** while the RELIABLE INCOME is but **£2,000,** leaving a DEFICIT of about **£4,000** to be raised annually from FRESH SOURCES.

The Committee therefore MOST EARNESTLY PLEAD FOR HELP to enable them to raise this deficit, and to provide for the daily-increasing number of Patients.

### ENLARGEMENT OF THE NURSES' HOME.

Upwards of **£5,000** is also urgently needed to provide more Ward accommodation for the constantly increasing number of patients and for an enlargement of the Nurses' Home.

CONTRIBUTIONS to both the General and the Building Funds will be thankfully received by the Hospital Bankers, Messrs. COCKS, BIDDULPH & Co., Charing Cross, S.W., or at the Hospital by

ARTHUR WATTS, *Secretary.*

### LONDON LOCK HOSPITAL AND RESCUE HOME

**HARROW ROAD, LONDON, W.**

Patron: HIS MAJESTY THE KING.

**NO LETTERS REQUIRED.**

**OVER 200 BEDS.**

**£2,000 Required before the Close of the Year.**

**The only Institution** of the kind in this or any other country with Hospital and Home combined.

Subscriptions and Donations will be gratefully received by the Treasurers, Lord Kinnaird, 1 Pall Mall East, S.W.; J. F. W. Deacon, Esq., 20 Birchin Lane, E.C.; or The Secretary, Harrow Road.

### IRISH DISTRESSED LADIES FUND.

**Patron—**

**HER MAJESTY QUEEN ALEXANDRA.**

**Executive Committee:**

*President—*
H.R.H. the PRINCESS LOUISE, DUCHESS OF ARGYLL.

*Vice-President—*
The MARCHIONESS OF WATERFORD.

*Chairman*—The Rt. Hon. the EARL OF ERNE, K.P.
*Deputy Chairman*—LT.-GENERAL R. W. LOWRY, C.B.
*Hon. Treasurer*—H. H. PLEYDELL BOUVERIE, Esq.
*Bankers*—Messrs. BARCLAY & CO., 1 Pall Mall East, S.W.

*Manageress—*
Work Depôt—Miss CAMPBELL, 411 Oxford Street, W.

*Secretary—*
GENERAL W. M. LEES, 411 Oxford Street, London, W.

The COMMITTEE APPEAL VERY URGENTLY for FUNDS for the relief of Ladies who depend for their support on the proceeds of Irish property, but who, owing to the non-receipt of their incomes from causes beyond their control, have been reduced to absolute poverty, and from old age and infirmity are unable to work.

**Office and Work Depôt—411 OXFORD STREET, W.**

### ASSOCIATION FOR THE ORAL INSTRUCTION OF THE DEAF AND DUMB.

Training College for Teachers of the Deaf on the German, or Pure Oral System, and School for Children (certified).

11 FITZROY SQUARE, LONDON, W.

**Patrons—THEIR MAJESTIES THE KING AND QUEEN.**

The Objects of the Association are—
(1) **To promote** the Pure Oral Instruction of the Deaf and Dumb by **Lip Reading** and **Articulate Speech**, to the **rigid exclusion** of the Finger Alphabet and all Artificial Signs.
(2) **To train qualified Teachers on this System** for public and private work.
(3) **To maintain a Normal School** for Instructing Deaf and Dumb Children of all Classes and denominations.

For all particulars apply to the Director, William Van Praagh, at the above address. Lip Reading Taught to Children and Adults who are Incurably Deaf. Public Lessons every Wednesday afternoon at Three o'clock, except during vacations.

**DONATIONS AND ANNUAL SUBSCRIPTIONS MOST URGENTLY REQUIRED.**

Bankers: Messrs. Robarts, Lubbock, and Co., 15 Lombard Street, E.C.

Orphan Working School. Founded 1758. Maintains and Educates 500 Fatherless Children.

### CHRISTMAS GIFTS

AND NEW

**Annual Subscriptions**

ARE MUCH NEEDED BY THE

### ORPHAN WORKING SCHOOL.

Senior School: MAITLAND PARK, N.W.
Junior School (Alexandra Orphanage): HORNSEY RISE, N.
Convalescent Home: HAROLD ROAD, MARGATE.

Patrons:
HIS MAJESTY THE KING.
HER MAJESTY QUEEN ALEXANDRA.
President:
H.R.H. The Prince of Wales, K.G.
Treasurer:
Sir Horace B. Marshall, M.A., LL.D., J.P.

**The Charity is not endowed, but depends upon Voluntary Contributions.**

Please help this most necessary work.
One Thousand new Annual Subscriptions are urgently needed.
Two votes at each half-yearly Election for every Guinea subscribed.
Information will be gladly given by the Secretary, to whom contributions should be sent.
ALEXANDER GRANT, Secretary.
Offices:—73 Cheapside, E.C.
Bankers:
The London Joint Stock Bank, Princes Street, E.C.

### Royal Maternity Charity of London.

**Patron: H.M. THE QUEEN.**

This Charity was instituted in 1757 to give Maternity Nursing and Medical Attendance to poor married women in their own homes, gratis. Over **3,000** are so helped annually. The Funds are greatly in debt, and

### HELP IS URGENTLY NEEDED

to continue the good work. Donations and Subscriptions thankfully received by the Treasurer, the Rt. Hon. Lord Avebury, or the Secretary, 31 Finsbury Square, E.C.

### THE CANCER HOSPITAL (Free)

**(Founded 1851),**

**FULHAM ROAD, LONDON, S.W.**

**The only Special Hospital in London for the treatment of Cancer.**

NO LETTERS NECESSARY.

**FUNDS URGENTLY NEEDED for the General Expenses and the Research Department.**

*Secretary,* FRED. W. HOWELL.

### LONDON ORPHAN ASYLUM, WATFORD.

**Under the Patronage of Their Majesties THE KING and THE QUEEN, T.R.H. THE PRINCE and PRINCESS OF WALES.**

*Instituted* 1813, *for the Maintenance and Education of Boys and Girls of Respectable Descent from every part of the British Empire.*

**Increased support is much needed.** While **£15,000** is required each year, **only £1,600** is assured, leaving **£13,400** to be obtained from **voluntary sources.** Over **6,570** Orphan Children have been benefited from all parts of the Empire, and nearly **500** such Children, representing all professions and trades, are now in the School. Benefits are dispensed in a truly national spirit, and the Managers hope for a correspondingly wide and liberal support.

Annual Subscriptions (from 10s. 6d.), Life Subscriptions (from £5 5s.), and Donations will be gladly received.

*Bankers*: GLYN, MILLS & Co., 67 Lombard Street.
Office, 21 Great St. Helen's, E.C.

E. H. BOUSFIELD, *Treasurer and Chairman.*
ARTHUR P. BLATHWAYT, *Deputy-Chairman.*
HENRY C. ARMIGER, *Secretary.*

### CITY OF LONDON LYING-IN HOSPITAL,

**CITY ROAD, E.C. (Instituted 1750).**

PATRON—The Right. Hon. THE LORD MAYOR.
TREASURER—A. J. ROBARTS, Esq. BANKERS—MESSRS. ROBARTS, LUBBOCK, AND CO.

The Committee earnestly appeal for **DONATIONS TOWARDS THE £25,000 REQUIRED FOR REBUILDING** the old portion of the Hospital, which has become absolutely necessary. Also for **NEW ANNUAL SUBSCRIPTIONS.**

Temporary premises for In-patients, 228 Old Street. Patients delivered last year, 3,100.

R. A. OWTHWAITE, Secretary.

**Established 1882.**

### THE HAMPSTEAD GENERAL HOSPITAL.

PATRON—H.R.H. The PRINCESS CHRISTIAN of SCHLESWIG-HOLSTEIN. PRESIDENT—Sir HENRY HARBEN, D.L., J.P.

VICE-PRESIDENTS—The Rt. Hon. The EARL of MANSFIELD; Sir SPENCER MARYON-WILSON, Bart., of Eastborne; EDWARD BOND, Esq., M.P.; J. S. FLETCHER, Esq., J.P.; WILLIAM S. GARD, Esq.; Colonel G. G. GORDON, C.B., C.V.O.

TREASURER—W. F. MALCOLM, Esq. BANKERS—LONDON and SOUTH WESTERN BANK (Hampstead Branch).

THIS HOSPITAL is **FREE** to the Poor of Hampstead, Highgate, and adjacent districts—including Gospel Oak, Kentish Town, Kilburn, and Hendon—with a few paying beds at charges from **12s.** to **£5 5s.** per week.

**1904—to November 30.**

| | |
|---|---|
| Accident and Emergency Cases admitted | 182 |
| Ditto Minor Cases treated | 560 |

| | |
|---|---|
| **GENERAL FUND.**—Average Annual Deficit on Ordinary Income | £550. |
| **BUILDING FUND.**—Balance required | £12,000. |

**AN EARNEST APPEAL IS MADE FOR DONATIONS AND NEW ANNUAL SUBSCRIPTIONS.**

GEORGE WATTS, Secretary.

### NORTH-WEST LONDON HOSPITAL

KENTISH TOWN ROAD.

ESTABLISHED 1878.

Patron—H.R.H. the PRINCESS CHRISTIAN.

President—The Most Hon. the MARQUIS CAMDEN.
Treasurer—GEORGE HERRING, Esq.
Chairman—The Right Hon. LORD RATHMORE.

This Hospital has 50 Beds, 15 of which are reserved for SICK CHILDREN. It is the only Institution of the kind in this densely-populated district. Last year there were 615 In-patients and 44,465 attendances of Out-patients.

The present income from Annual Subscriptions is less than £700, and the yearly expenditure over £4,500. There is consequently a large deficiency to be met each year by special benefactions. Help is earnestly solicited.

**"Well worthy of Support."**
See Report of King Edward's Hospital Fund.

ALFRED CRASKE, Secretary.

### SAMARITAN FREE HOSPITAL

### FOR WOMEN,

### MARYLEBONE ROAD, N.W.

**Dependent upon Voluntary Benevolence for Support.**

ENTIRELY FREE to poor women suffering from diseases peculiar to their sex.

**GREATLY IN NEED OF FUNDS TO MEET CURRENT EXPENSES.**

***£15,000 still required for New Buildings.***

**Contributions (particularly Annual Subscriptions and Bequests) earnestly solicited.**

GOVERNORSHIP: Annual Subscription of £2 2s. 0d. LIFE GOVERNORSHIP: Donation of £25.

Bankers: Sir SAMUEL SCOTT, Bart., AND Co., 1 Cavendish Square, W. W. GUNTRIP KING, Secretary.

### THE HOSPITAL AND HOME FOR INCURABLE CHILDREN,

### NORTH COURT, HAMPSTEAD, N.W.

*Patrons*—H.R.H. The PRINCESS CHRISTIAN; H.R.H. The DUCHESS OF CONNAUGHT.
*President*—H.R.H. The DUKE OF CONNAUGHT AND STRATHEARN, K.G.
*Hon. Treasurer*—V. BISHOP, Esq. *Hon. Sec.*—HENRY SEWELL, Esq.
*Bankers*—Messrs. HOARE, 37 Fleet Street, E.C.; PARR'S BANK, Bartholomew Lane, E.C.

This Institution, founded in 1875, was the first of its kind in the Metropolis, and, it is believed, in the United Kingdom. It was established with the object of providing for the maintenance, care and medical treatment of Children (up to the age of sixteen) suffering from Chronic or Incurable Complaints of an aggravated character. It combines the advantage of a Home and a Hospital.

Over **200** Children have been received, and there are **Thirty** cots. A small Weekly payment is required for each child; but the expenses of such a charity are necessarily very large, and the Committee **appeal earnestly for Donations and new Subscriptions,** which are much needed. New premises have been recently acquired at a large outlay, to meet which funds are urgently required. Visiting hours 3 to 5 p.m. daily. **Miss FORSTER, Matron.**

### Miller Hospital & Royal Kent Dispensary,

### GREENWICH ROAD, S.E.

**Patron - - HIS MOST GRACIOUS MAJESTY THE KING.**

*President*—The Rt. Hon. THE EARL OF DARTMOUTH. *Treasurer*—I. HAMILTON BENN, Esq.

The Institution is situated in a poor and densely-populated neighbourhood on the borders of Deptford and Greenwich. It is **STILL** the only General Hospital south of the Thames, with the exception of St. Thomas's and Guy's.

**In-Patients treated last year - - - - - 268.**
**Out-Patients „ „ - - - - - 19,413.**

**ANNUAL SUBSCRIPTIONS AND DONATIONS NEEDED FOR THE GENERAL FUND.**

The Enlargement of the Hospital is imperative, and the **Committee Urgently Appeal for Contributions to the Extension Fund. £5,000 will enable 20 Beds to be added.**

JAMES MARKS, *Secretary.*

### LONDON FEVER HOSPITAL,

### LIVERPOOL ROAD, ISLINGTON, N.

**THE HOSPITAL RECEIVES NO HELP FROM THE RATES.**

Over 82,000 persons, mostly suffering from Scarlet Fever or Diphtheria, have been treated here since 1802.

Patients pay about a fourth of their cost, the balance falling upon the funds of the Institution.

THE BENEVOLENT ARE EARNESTLY ASKED FOR ADDITIONAL HELP IN THE WORK OF CHECKING THE SPREAD OF SCARLET AND OTHER INFECTIOUS FEVERS. The Hospital receives no help from the rates.

Donors of £10 10s. 0d. in one sum are Governors for life. Annual Subscribers of £1 1s. 0d. or more are Governors on payment of the second year's subscription, and as long as they continue to subscribe.

Servants of Governors, and certain employés of subscribing firms, clubs, and hotels, are promptly removed and treated free of all charge.

DONATIONS and SUBSCRIPTIONS will be gratefully received by the Secretary at the Hospital.

Bankers: PRESCOTT AND Co., 50 Cornhill, E.C. W. CHRISTIE (Major), Secretary.

### BRITISH ORPHAN ASYLUM, SLOUGH.

*Patron*—**HIS MAJESTY THE KING.** *Patroness*—**HER MAJESTY QUEEN ALEXANDRA.**

Established in 1827 for the MAINTENANCE and EDUCATION of ORPHAN CHILDREN from all parts of the British Empire, of all denominations, whose parents were once in prosperous circumstances. Boys and Girls are admitted by Election, Purchase, and Presentation between the ages of 7 and 12, and are retained until 15.

The Committee EARNESTLY APPEAL for ANNUAL SUBSCRIPTIONS and DONATIONS, which are URGENTLY NEEDED, this old-established National Charity being Dependent on Voluntary Aid.

*Offices*—27 Clement's Lane, E.C. J. F. W. DEACON, Treasurer. CHARLES T. HOSKINS, Secretary.

Annual Subscription for One Vote, 10s. 6d.; for Two Votes, £1 1s. Life Donation for One Vote, £5 5s.; for Two Votes, £10 10s. Life Presentation, £350.

*Bankers*—Messrs. Williams Deacon's Bank, Limited, 20 Birchin Lane, E.C.

"Help the Fatherless and Orphan."

### THE ROYAL ORPHANAGE, WOLVERHAMPTON.

*FOUNDED 1850.*

For the Maintenance, Education, and Clothing of Necessitous Orphan Children from all parts of the Kingdom.

**SUPPORTED BY VOLUNTARY CONTRIBUTIONS.**

Patrons—HIS MOST GRACIOUS MAJESTY THE KING; HER MOST GRACIOUS MAJESTY QUEEN ALEXANDRA.
Vice-Patrons—T.R.H. The PRINCE and PRINCESS OF WALES, and H.R.H. PRINCESS HENRY OF BATTENBERG.

**Objects and Advantages of the Institution.**

It affords the advantages of Home and Education to Fatherless Children of either sex who are destitute of the means of support, and who are retained in the Institution till fifteen years of age.

Orphans are eligible as Candidates between the ages of seven and eleven years; the Elections taking place half-yearly (April and October).

Over Three Hundred Children are now in the Establishment, which is capable of receiving a much larger number when sufficient Funds are obtained for their support. The Institution has accommodation for Four Hundred Children.

The Subscribers have the power of nominating Candidates, and the right of voting at all Elections, according to the amount of their subscriptions.

**Annual Subscription** for **Two** Votes at each Election, **10s. 6d.** For **Four** Votes, **£1 1s.** For **Eight** Votes, **£2 2s.**, and so on in proportion to the amount given.

**Life Subscriptions** for **Two** Votes at each Election, **£5 5s.** For **Four** Votes, **£10 10s.**, and so on in proportion to the amount given.

Children, eligible by the rules, can be admitted, irrespective of election, on the payment in one sum of **150 Guineas** (if between the ages of **7** and **9**); **100 Guineas** (if between the ages of **9** and **11**).

WALTER HAMBLETT, Secretary.

---

### Metropolitan Convalescent Institution.

FOUNDED 1840.

*Patrons*—His Most Gracious Majesty the KING and Her Majesty QUEEN ALEXANDRA.
*President*—The Rt. Hon. Viscount PORTMAN. *Chairman of the Board of Management*—M. O. FITZ-GERALD, Esq.

### — BRANCHES. —

**WALTON-ON-THAMES.** (250 Beds for Adults.)

**BROADSTAIRS.** (118 Beds for Children, with Wards for Surgical Cases.)

**BEXHILL-ON-SEA.** (125 Beds for Adults.)

**ST. LEONARDS-ON-SEA.** (Temporary Home, 50 Beds for Women.)

Nearly **7,000 Patients** are admitted to the Homes every year, either upon their discharge from Hospital or after illness in their own homes, entirely **Free of Charge.** The Board of Management **APPEAL** very earnestly for further **ANNUAL SUBSCRIPTIONS and DONATIONS.** The maintenance of the Four Homes costs about **£13,000** a year, for nearly the whole of which the Institution is dependent upon voluntary contributions.

TREASURERS—The Right Hon. LORD HALIBURTON, G.C.B., and JAMES S. STRANGE, Esq.
Office:—32 Sackville Street, W. ALEX. HAYES, Secretary.

---

### FIELD LANE RAGGED SCHOOLS & REFUGES.

### THE DESERVING UNEMPLOYED

Are being **HELPED DAILY** by this Institution. Hot Dinners three days a week to 100 poor Children. Food Distributed daily to about 100 Families. Dinner provided for 900 persons on Boxing Day. Provisions for Christmas Dinner for 1,500 Families.

**THE COMMITTEE EARNESTLY APPEAL FOR FUNDS.**

*Treasurer*—W. A. BEVAN, Esq., 54 Lombard Street, E.C.
*Secretary*—PEREGRINE PLATT, Vine Street, Clerkenwell Road, E.C.

---

### THE MARY WARDELL CONVALESCENT HOME FOR SCARLET FEVER, Stanmore.

PATRONESSES: HER MAJESTY THE QUEEN. H.R.H. THE DUCHESS OF ALBANY.

**TWENTY-FIVE YEARS' WORK.**

In the Year 1879 the first steps were taken towards founding a Home for the reception of Convalescents from a special illness which debarred them from admission to all existing Convalescent Homes. It has now been at work for twenty years, after five years' preliminary hard work to raise funds, purchase a freehold, and build. More than 4,000 Patients have benefited by it. The Foundress is now in her 73rd year, and appeals urgently for help to relieve the Home of a debt of **£600** and increase the annual support.

SUBSCRIPTIONS and DONATIONS may be sent to Messrs. BARCLAY and Co., and to Miss MARY WARDELL, Hon. Secretary, Stanmore, Middlesex.

### THE HOSPITAL FOR SICK CHILDREN

*Established in 1852.*

### Great Ormond Street, Bloomsbury, W.C.

### THE FIRST CHILDREN'S HOSPITAL IN THE BRITISH EMPIRE.

***Convalescent Branch—CROMWELL HOUSE, HIGHGATE, N.***

**Patrons.**

**THEIR MAJESTIES THE KING AND QUEEN.**

Vice Patrons.

THEIR ROYAL HIGHNESSES THE PRINCE AND PRINCESS OF WALES.

HER ROYAL HIGHNESS THE DUCHESS OF FIFE.

HER ROYAL HIGHNESS THE PRINCESS CHRISTIAN.

President—THE DUKE OF FIFE, K.T. Treasurer—J. F. W. DEACON, Esq.

Chairman—ARTHUR LUCAS, Esq. Vice-Chairman—JOHN MURRAY, Esq

**£1,000 will endow a Bed for ever in memory of a relative or friend.**

**200 Beds** at Great Ormond Street.

**52 Beds** at Cromwell House.

**Over 850,000** Sick Children have been treated during the past 52 years.

**Every day £20** has to be raised to keep open the wards of the **oldest and largest Children's Hospital** in the British Empire, and

**NEW ANNUAL SUBSCRIPTIONS**

URGENTLY NEEDED.

E. STEWART JOHNSON, Secretary.

Printed by SPOTTISWOODE & CO. Ltd., New Street Square, E.C.; and Published by the Proprietors, THE SCIENTIFIC PRESS, LIMITED, at 28 & 29 Southampton Street, Strand, London, W.C.

## Figures and Tables

**Figure f1:**